# Assessing morphological preservation of gastrointestinal parasites from fecal samples of wild capuchin monkeys (*Cebus imitator*) stored in ethanol versus formalin

**DOI:** 10.1038/s41598-024-53915-2

**Published:** 2024-02-13

**Authors:** Joelle K. Hass, Megan C. Henriquez, Jessica Churcher, Hadjira Hamou, Suheidy Romero Morales, Amanda D. Melin

**Affiliations:** 1 Department of Anthropology and Archaeology, University of Calgary, Calgary, AB Canada; 2 Host Parasite Interactions Network, University of Calgary, Calgary, AB Canada; 3 Department of Anthropology, The Graduate Center, City University of New York, New York, NY USA; 4 The New York Consortium in Evolutionary Primatology (NYCEP), New York, NY USA; 5 Área de Conservación Guanacaste, Guanacaste, Costa Rica; 6 Alberta Children’s Hospital Research Institute, University of Calgary, Calgary, AB Canada; 7 Department of Medical Genetics, University of Calgary, Calgary, AB Canada

**Keywords:** Microbiology, Diseases, Infectious diseases, Ecology, Ecological epidemiology

## Abstract

The copromicroscopic identification of gastrointestinal parasites is a common, cost-effective method vital to understanding host-parasite interactions. However, its efficacy depends on effective preservation of the samples. In this study, we compare the preservation of ethanol and formalin preserved gastrointestinal parasites collected from a wild population of Costa Rican capuchin monkeys (*Cebus imitator*). Fecal samples were collected, halved, and stored in either 10% formalin or 96% ethanol at ambient temperature, then microscopically screened for the presence of parasites. Parasites were morphologically identified and rated based on their preservation using a newly developed rubric. We identified more parasitic morphotypes in formalin-preserved samples but found no difference in the number of parasites per fecal gram (PFG) between mediums. There was no difference in the PFG of two most prevalent parasite morphotypes, *Filariopsis barretoi* larvae and Strongyle-type eggs, and while *Filariopsis* larvae were better preserved in formalin, strongyle eggs showed no preservation difference between mediums. Our results support the suitability of both ethanol and formalin for morphological parasite identification in samples stored over 1 year, describe the morphological changes and challenges associated with parasite degradation, and highlight the potential for future studies to use both morphological and molecular methods in non-invasively collected samples.

## Introduction

The morphological identification of gastrointestinal parasites using copromicroscopy has laid the foundation in the field of veterinary parasitology for several decades and continues to be the gold standard in many diagnostic and clinical settings^1^. Microscopy is cost-effective and provides rapid results, but requires observers to be highly trained, limiting the number of experts in the field^2^. Even with years of training and experience, veteran parasitologists face significant challenges when morphologically identifying parasites, as closely related taxa share many characteristics and may be visually indistinguishable in egg and larval forms^3^. Additionally, host species, host diet, and environmental factors can produce variation in the morphological characters traditionally used to identify species^1^. To accommodate for the uncertainty in species-level identifications, researchers will often keep taxonomic assignments broad, at either the genus, family, or order level (e.g., “strongyle-type” egg for thin-shelled, ovular, embryonated egg which could be in the genus *Strongyloides* (threadworm), *Necator*, or *Ancylostoma* (hookworms)). These broad-level taxonomic identifications provide a glimpse into the composition of parasitic communities within hosts, but also underestimate the true taxonomic, genetic, and biological diversity of these communities^2^.

Advances in molecular parasitology in the last two decades have revealed new insights into parasite genetic diversity and evolutionary history through the development of DNA and protein-based methods such as enzyme-linked immunosorbent assay (ELISA), loop mediated isothermal amplification (LAMP), shotgun metagenomics, deep amplicon sequencing, and others^4–7^. Molecular analyses have allowed researchers not only to distinguish between morphologically similar species, but to develop nematode phylogenies that promote our understanding of parasite evolutionary history and host-parasite-environment relationships^8,9^.

Despite new molecular methods, microscopy remains important to the field of parasitology. Thus, optimization of parasite preservation for morphological identification remains a priority, and preservation methods amenable to molecular as well as morphological investigation are ideal^5,10,11^. The typically preferred preservative for morphological identification is formalin, which has been used as an embalming fluid for centuries^12,13^. Formalin forms amino acid cross-links between proteins in tissues, creating a matrix that prevents autolysis and putrefaction, thus maintaining tissue form, however, these cross-links cause DNA fragmentation, impeding genetic analyses^12,14,15^. Additionally, formalin is toxic and needs to be handled carefully to prevent inhalation and skin contact. Preservation of fecal samples in ethanol (70–96%) is less common in morphological parasite studies, but is less toxic, easier to source, and has been successfully used in coprological studies^9^. Ethanol may be especially useful for molecular studies, as it has been shown to maintain stable levels of DNA during long-term storage^6^. However, its suitability for morphological analyses has been questioned as it dehydrates tissues, resulting in potentially degraded, brittle, and morphologically altered specimens^16,17^. Surprisingly, few studies have directly and rigorously compared the effect of ethanol versus formalin as storage mediums on parasite morphology.

This study aims to evaluate the preservation and morphological properties of gastrointestinal parasites found in fecal samples collected from a population of wild capuchin monkeys (*Cebus imitator*). Upon initial deposition, samples were halved such that one part was stored in 96% ethanol and the other in 10% buffered formalin, providing a comparison of the same samples, collected at the same time, and stored for approximately the same amount of time. The results of this study will provide information on the types of biases, if any, created by different storage media, and inform future studies seeking to explore both the morphological and genetic diversity of gastrointestinal parasites in fecal samples.

## Materials and methods

### Study site and subjects

We collected fresh fecal samples from a population of wild, habituated capuchin monkeys inhabiting the Sector Santa Rosa field site in the Área de Conservación Guanacaste, Costa Rica (10.82049 lat.; 85.62813 long.). We sampled 20 individuals from five habituated social groups, including both males and females, and individuals spanning a wide range of ages (Supplementary Table S1).

### Sample collection and coproscopy

We collected samples in July and August of 2021. Fresh fecal samples were collected immediately following defecation and partitioned into 2 halves. Approximately 2 g of the sample was stored in a sterile 15 ml tube containing 6 ml of 96% ethanol, and 2 g was placed in a sterile 15 ml tube containing 10 ml of 10% buffered formalin. The two halves were fully submerged and gently agitated within the solvents to assist with permeation of the sample. We collected and divided 21 fecal masses from 20 unique and individually identifiable capuchin monkeys, resulting in 21 paired samples, or 42 unique samples overall. The two stored halves of each sample were given unique, consecutive sample numbers, so that each “sample” refers to one half of the fecal mass stored in a particular preservation medium (Supplementary Table S1). Samples were stored at ambient temperature at the field site before being shipped to and stored at the Kutz Veterinary Parasitology Lab, Faculty of Veterinary Sciences, University of Calgary, Canada, where they were stored at ambient temperature between 8 and 19 months prior to microscopic analysis.

Samples were initially processed using a modified Wisconsin sedimentation technique. We first separated the solid sample from the liquid preservation medium and weighed the solids to determine the fecal weight, then homogenized the sample with distilled water and strained it through a double-layered cheese cloth. Next, we centrifuged the resulting solution for 10 min at 1500 rpm before discarding the supernatant and homogenizing the pellet with 5–10 ml of distilled water. Finally, we distributed the pellet into a 6-well microscopy plate for screening. Samples were screened using an Olympus CKX53 microscope with an Olympus DP72 camera and were photographed using CellSens Standard 1.18 build 16686. Parasite species were identified using morphological characteristics described in previous studies, including internal and external organs and appendages for larvae, and shape, size, and shell thickness for eggs^18–20^.

### Parasite degradation grading scale

**Figure 1:**
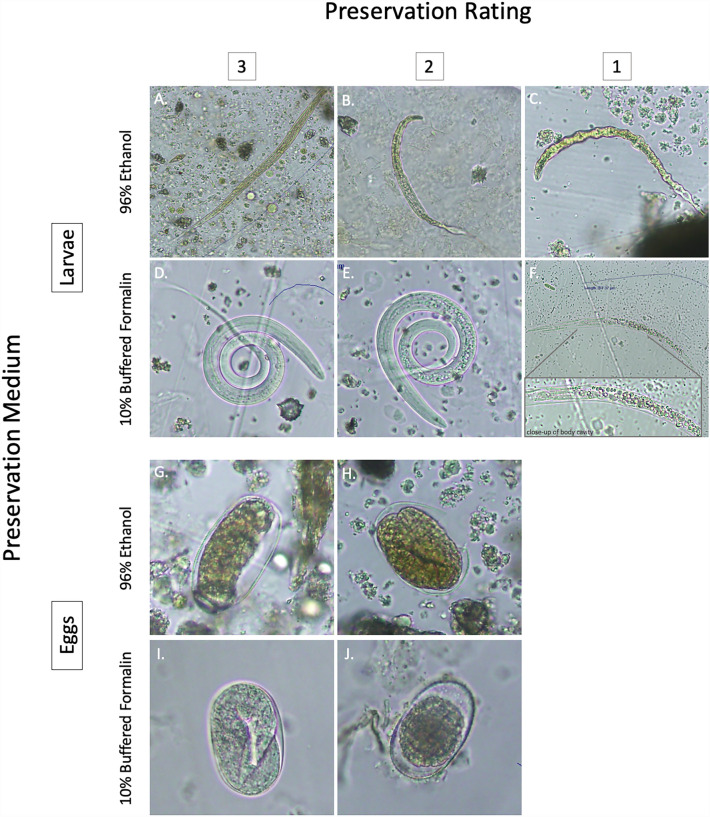
Examples of parasitic larvae (**A**–**F**) and eggs (**G**–**J**) identified in capuchin monkey (*Cebus imitator*) fecal samples and their state of preservation (preservation score) in either 96% ethanol or 10% buffered formalin. Larvae with a preservation score of 3 (**A**,**D**) show very little damage to their cuticle and have no or relatively minor alterations to their internal and external structures. Larvae with a preservation score of 2 (**B**,**E**) show moderate damage to the cuticle, as well as the internal and external organs. Larvae with a preservation score of 1 (**C**,**F**) show significant damage to the cuticle and internal/external organs and are often difficult to morphologically identify due to changes in and degradation of internal structures and overall form (i.e., size, shape, characteristic organs and appendages, etc.). Eggs with a rating of 3 (**G**,**I**) are clear, intact, and show minimal or no signs of damage, while eggs with a rating of 2 (**H**,**J**) show minor to moderate damage of the eggshell and may exhibit uncharacteristic changes in size or shape. We did not identify any poorly preserved eggs warranting a score under a 2 in these samples, and so the scale for eggs preserved in either ethanol or formalin is 3–2, instead of 3–1 for larvae. However, an example of a poorly preserved egg is available in Supple Fig. S2.

Three-point grading scales were created for both ethanol and formalin separately to describe the extent of parasite egg and larval degradation in our samples because the process differed between the two preservatives (Fig. 1). All parasites were graded by the same researcher (JKH), to minimize bias from subjectivity in the visual rating scale. For larvae, a three-point rating was given to well-preserved larvae with a fully intact cuticle, visible internal structures, and identifiable, morphologically unaltered external features (Fig. 1A, an ethanol “3”; Fig. 1D, a formalin “3”). A two-point rating was given to larvae which had degradation of either the cuticle (shrinking, puckering, thinning, increased opacity, etc.) or the internal structures (change in shape, clarity, obscured by cuticle or other features, etc.) that partially interfered with morphological identification (Fig. 1B, an ethanol “2”; Fig. 1E, a formalin “2”). Larvae assigned one point were heavily degraded and difficult, if not impossible, to morphologically identify due to significant changes in the cuticle and internal/external structures (Fig. 1C, an ethanol “1”; Fig. 1F, a formalin “1”). Typically, larvae with ratings of 1 would have their internal structures completely obscured by either cuticle thickening/deformation in ethanol-preserved samples or the overwhelming presence of ‘bubbles’ within the body cavity in formalin-preserved samples. Eggs were graded on the intactness of their shell and the visibility of the embryo or larva within the egg. Eggs given a score of 3 were clear, of the appropriate shape and size for the taxon, contained visible embryos/larvae, and had a continuous, unobstructed, unbroken shell. Eggs given a score of 2 had minor deformations in the shell (i.e., dents, breaks, increased thickness, and opacity, etc.) which may or may not have impacted the developing parasite within. We did not consider stage of embryonic development when grading eggs. Eggs were relatively well preserved in the samples used for this study, and so we did not assign any eggs in this study a grade lower than 2. However, badly preserved eggs warranting a score of one were found in samples not included in this study and an example has been included for reference (Supplementary Fig. S1).

### Statistical analyses

Data were analyzed in R studio version 4.3.1. We first used linear models to test whether the number of days in storage affected the parasite morphotype diversity, parasites per fecal gram (PFG), and average parasite preservation rating. Morphotype diversity was defined as the number of morphologically distinct parasite taxa identified in a sample. All individual parasites in a sample were given a grade scale rating from one to three. The sample was then assigned an overall rating calculated as the average parasite preservation rating. We did not calculate average rating separately for eggs and larvae in a given sample. Our small, zero-inflated non-normally distributed dataset did not meet the statistical requirements for generalized linear mixed models, so we used Wilcoxon-Signed Rank tests to compare the morphotype diversity, parasite PFG, and average preservation rating between mediums. We also compared the prevalence and PFGs of our two most common parasites, *Filariopsis* larvae (FPFG) and strongyle-type eggs (SPFG) between mediums. Four formalin samples were analyzed before standardization of the grading scale, and as such were not included in the preservation rating analyses. Thus, comparison of average parasite preservation ratings between mediums included 17 pairs, while comparisons of morphotype diversity, PFG, FPFG, and SPFG between mediums included all 21 pairs.

### Ethical approval

The animal study was approved by Animal Care Committee of the University of Calgary (ACC protocol AC19-0167) and the Institutional Animal Care and Use Committee of Queens College, City University of New York (CUNY; IACUC protocol 195). The study was conducted in accordance with the local legislation and institutional requirements.

## Results and discussion

### Parasite morphotype diversity

**Figure 2:**
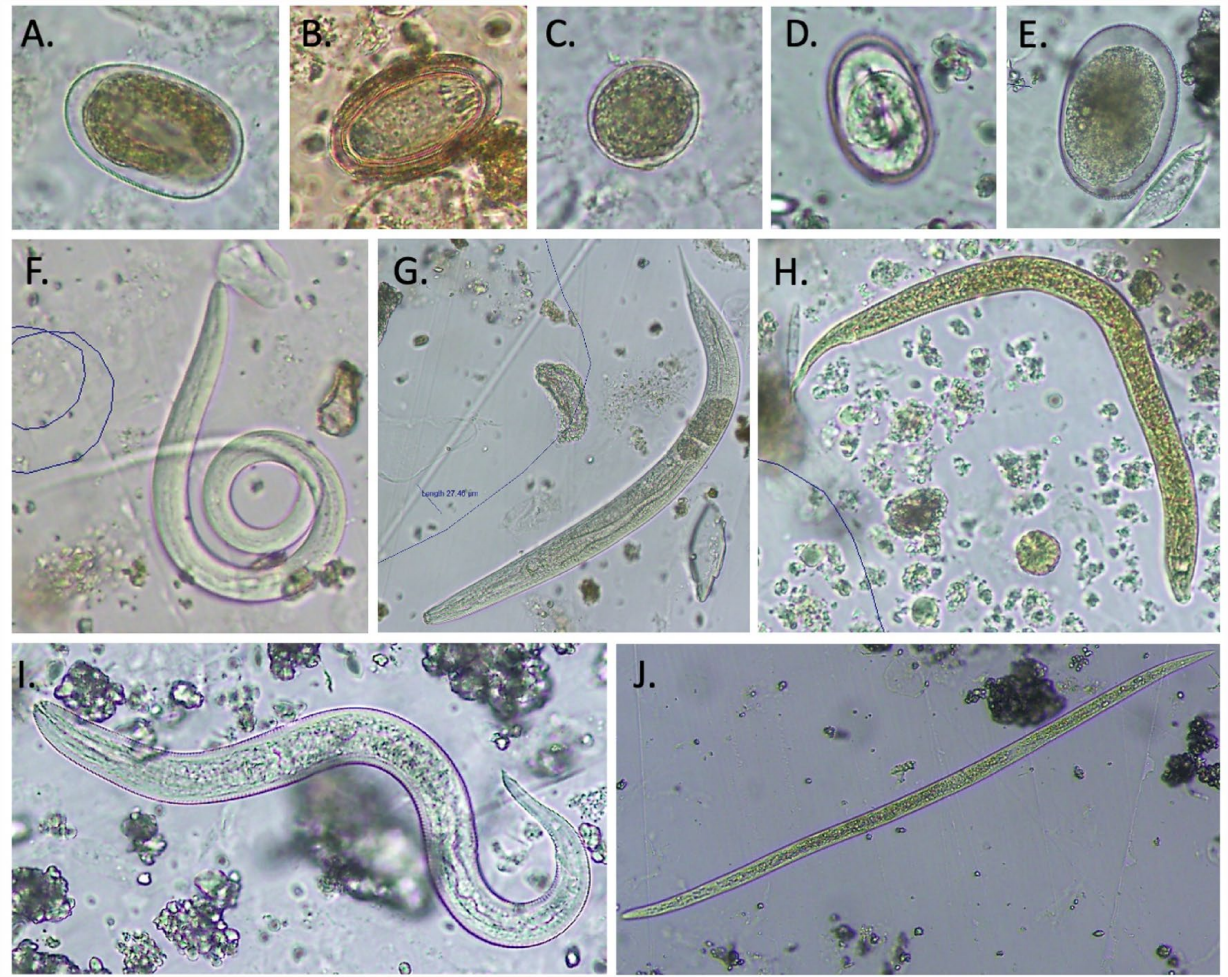
Gastrointestinal parasites collected from a wild population of capuchin monkeys (*Cebus imitator*) living from Costa Rica. (**A**) Strongyle-type egg (~ 57 × 36 μm), (**B**) Acanthocephalan egg (possibly *Prosthenorchis *sp.) (~ 62 × 37 μm), (**C**) Coccidian oocyst (possibly *Eimeria *sp.) (~ 30 × 30 μm), (**D**) unidentified egg (38 × 24 μm), (**E**) unidentified strongyle-type egg (large) (~ 93 × 61 μm), (**F**) *Filariopsis *sp. larva (381 μm), (**G**) unidentified larva morphotype 1 (~ 380 × 27 μm), (**H**) unidentified larva morphotype 2 (~ 235 μm) (**I**) unidentified larva morphotype 3 (~ 308 μm), (**J**) unidentified larva morphotype 4(possibly filariform *Strongyloides *sp.) 551 μm.

We found an average of 4.5 ± 1.9 parasitic morphotypes per fecal sample (range 1–9), with the two most prevalent morphotypes being *Filariopsis* larvae (42/42 samples) and strongyle-type eggs (possibly *Strongyloides cebus*; 36/42 samples). Other parasites include acanthocephalan eggs (possibly *Prosthenorchis *sp.), coccidian protozoan oocysts (possibly *Eimeria *sp.), and several nematode larval and egg morphotypes we were unable to identify with confidence (Fig. 2).

### Factors affecting parasite preservation

Samples were stored at room temperature in their respective preservation mediums for an average of 344.57 days (± 59.94; range 258–466 days). The latency from date of collection or the sample ‘age’, did not affect the number of parasitic morphotypes detected (F = 0.658, Adjusted R2 = − 0.008, 95% CI − 13.914 to 5.944, P = 0.422), parasites per fecal gram detected (F = 1.300, Adjusted R2 = 0.007, 95% CI − 0.138 to 0.038, P = 0.260), or average parasite preservation rating for either medium (Ethanol: F = 0.048, Adjusted R2 = − 0.053, 95% CI − 76.906 to 62.367, P = 0.829; Formalin: F = 0.031, Adjusted R2 = − 0.057, 95% CI − 364.466 to 307.969), suggesting that sample age does not significantly impact the patterns of preservation documented in each medium, at least for the range of sample collection dates analyzed in our study.

Parasites tended to have higher preservation ratings in formalin than in ethanol (Fig. 2A). We found more parasitic morphotypes in samples preserved in formalin compared to those preserved in ethanol (Wilcoxon signed rank test with continuity correction, V = 19.5, P = 0.012, 95% CI − 3.999 to − 0.500; Fig. 2B). However, samples preserved in formalin did not have more parasites per fecal gram than samples preserved in ethanol (Wilcoxon signed rank test with continuity correction, V = 67, P = 0.095, 95% CI − 89.499 to 7.499; Fig. 2C).

Our two most prevalent parasite morphotypes, *Filariopsis* larvae and strongyle eggs, were found in 100% and 85.7% of samples respectively, and their abundance did not differ significantly between preservation mediums (FPFG: Wilcoxon signed rank test with continuity correction, V = 68.5, P = 0.106, 95% CI − 87.499 to 7.99; SPFG: Wilcoxon signed rank test with continuity correction, V = 68, P = 0.272, CI − 2.499 to 0.499). *Filariopsis* larvae tended to be better preserved (i.e., clearer, cuticle more intact, no internal or external deformation) in formalin (Wilcoxon signed rank test with continuity correction, V = 9, P < 0.01, CI − 1.369 to − 0.985), while strongyle-type eggs showed no clear difference in preservation status between mediums, at least for the storage durations in this study—up to 19 months (Wilcoxon signed rank test with continuity correction, V = 42, P = 0.844, CI − 0.312 to 1.438).

While our data suggest that both formalin and ethanol are broadly viable options for medium-term preservation of gastrointestinal parasites, some differences in efficacy do seem to exist. Larvae in formalin samples tended to have clearer internal features and intact cuticles, whereas larvae in ethanol often appeared altered, especially *Filariopsis* larvae. The outer cuticle of *Filariopsis* larvae frequently appeared wrinkled and cracked, and in some extreme cases, constrained tightly around the internal structures, giving the entire larva the appearance of having been dehydrated (Fig. 1C). Severe wrinkling of the outer cuticle in ethanol-preserved larvae obstructed our view of both internal and external diagnostic features. This degradation may make morphological identification of larval stages more difficult, especially since the outer cuticle of different nematode species can have characteristic striations, spikes, or rings crucial to proper morphological identification^21^. Additionally, the wrinkling of the cuticle may obscure the view of diagnostic internal structures, such as the pharynx, genital primordium, or hindgut^21^. In contrast, most eggs and larvae preserved in formalin remained bright and translucent, with smooth cuticles and distinct internal features. However, degradation did occur in some larvae in a manner that seemed to affect the pseudocoelom first, then the body wall, and finally the cuticle. In formalin-preserved larvae that we described as ‘moderately preserved’ (a rating of 2), internal structures were partially obstructed by iridescent, granular, ‘bubbles’, visually similar to the effect of putting drops of oil on water. In poorly preserved larvae (a rating of 1), these ‘bubbles’ filled the entire body cavity, obstructing views of the buccal cavity, esophagus, and other structures (Fig. 1E). In a few extreme cases, it appeared as though the cuticle was breaking down as well, leaving behind a faint outline of what was presumably once, a whole larva (Fig. 1F).

**Figure 3:**
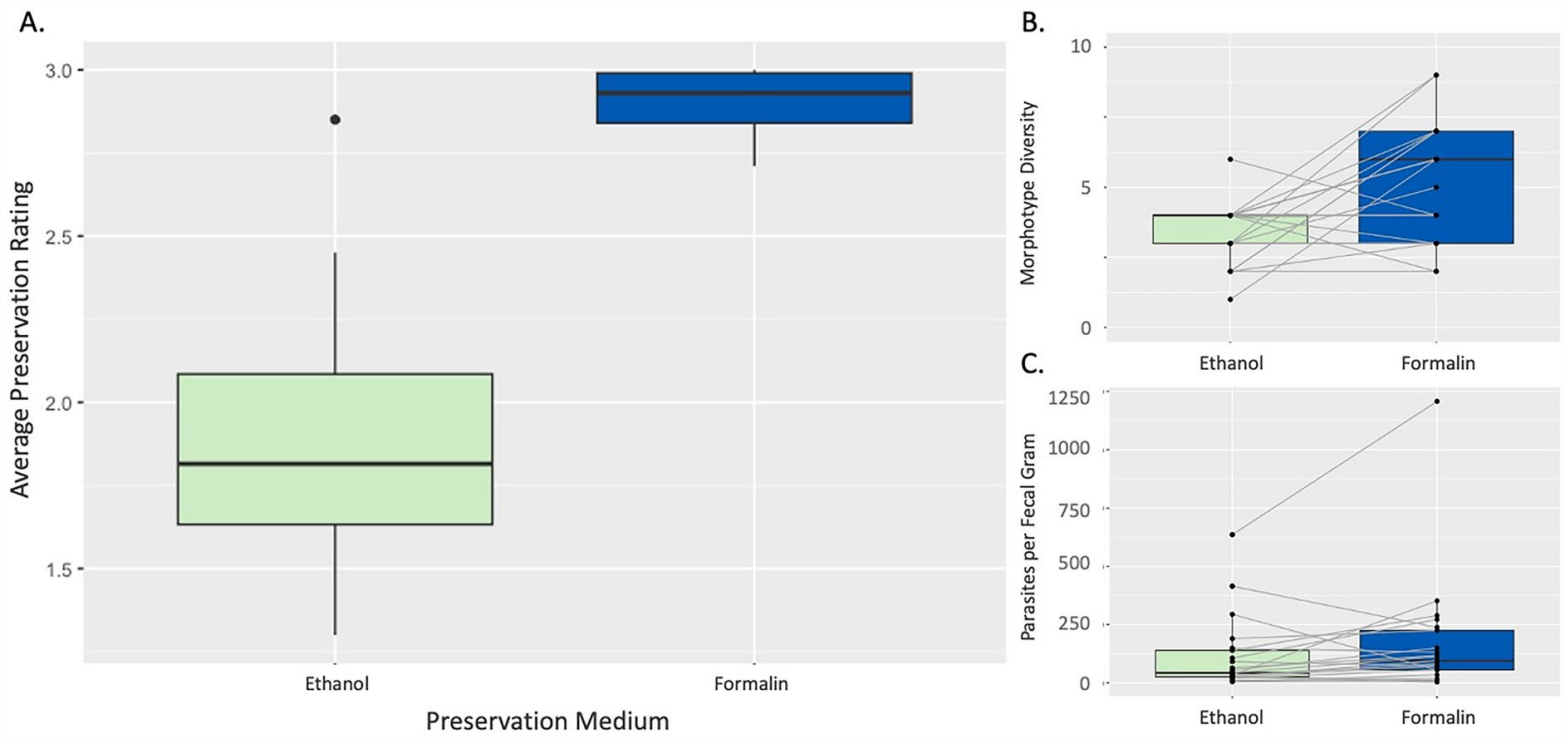
Average preservation rating, morphotype diversity, and parasite per fecal gram for gastrointestinal parasites in fecal samples collected from a wild population of capuchin monkeys (*Cebus imitator*) and stored in either 10% buffered formalin or 96% ethanol. Average parasite preservation ratings and morphotype diversity were significantly higher in formalin preserved samples when compared using Wilcoxon signed-rank tests.

The samples in this study were collected and fully submerged in the preservation medium immediately after defecation. Additional analyses also suggest that sample age, measured as the number of days between sample collection and screening, did not impact average parasite preservation rating in either ethanol or formalin for the ranges of sample age included in this study. The morphological changes witnessed in degrading larvae preserved in formalin were also distinct from those witnessed in ethanol-preserved larvae, as formalin-preserved larvae seemed to disintegrate from the ‘inside-out’, showing significant changes in the pseudocoelom occurring before changes in the body wall and cuticle. Larvae preserved in ethanol appeared to dehydrate from the ‘outside-in’, thickening and shrinking in the outer body layers (e.g., cuticle, body wall), which obstructed morphological identification. The differing characteristics of morphological degradation in larvae preserved in the two mediums suggest that different biochemical processes are at work and the observable changes are not due to pre-fixation processes such as autolysis, which we would expect to affect parasites similarly despite the preservative they were stored in (Fig. 3).

Overall, we found more well-preserved parasites in formalin samples, which allowed for detailed morphological examination of both internal and external structures. The differences in numbers of parasite morphotypes detected between mediums likely reflects variation in our ability to identify and distinguish between different larval morphotypes due to degradation. The number of morphotypes detected in our ethanol samples may be an underestimation due to our inability to detect subtle morphological characteristics hidden by a physically altered cuticle or body wall. Alternatively, our estimation of morphotype diversity in our formalin samples may be inflated due to the degradation process introducing ‘bubbles’ into the internal cavity and altering the appearance of diagnostic internal structures. Indeed, previous studies examining morphological changes in long-term formalin-fixed tissue found that microscopic changes in tissue integrity and architecture were more prevalent in tissue samples stored a minimum of 5 years when compared to fresh samples^15^, suggesting that a number of abiotic and chemical changes occurring over a prolonged period of time may lead to significant morphological changes in the sample. Regardless, the similarity in the number of parasites per gram of feces retrieved from samples preserved in either medium suggests that it is unlikely that a considerable number of eggs and larvae are degrading and disintegrating in ethanol, but that differences in morphotype richness may be due to our ability to morphologically identify and distinguish between different larvae^1^. However, morphological changes precipitated from preservation in a very high concentration of ethanol may be lessened if 70–80% ethanol was used instead^22,23^. Additionally, some of the dehydrating morphological effects of high concentration ethanol preservation may be reversed by rehydrating parasites in a solution of dimethyl sulfoxide (DMSO), disodium EDTA (ethylenediaminetetraacetic acid) and sodium chloride followed by water^24^. Future research may benefit from testing whether parasites are better suited for morphological examination at these concentrations, which should still permit successfully extracting DNA. Fortunately, eggs did not seem to be severely impacted by degradation in either medium and retained their diagnostic characteristics (size, shape, shell thickness, etc.) throughout the duration of this study.

Previous studies that have paired morphological and molecular parasite identification approaches typically separate individual samples into aliquots of ethanol or formalin and use the aliquot stored in the appropriate medium for either morphological or molecular analyses^2,6,9,10^. However, conducting morphological and molecular analyses on the same parasites can reveal important information regarding genetic diversity and cryptic species within hosts. Indeed, preliminary research suggests that a paired morphological and molecular parasite identification approach using ethanol preserved fecal samples has the potential to genetically identify parasites and other non-parasitic nematodes previously unidentified in the host using morphology alone (Henriquez et al. *In Review*). Collecting fecal samples in 96% ethanol and storing them at room temperature has been shown to be an effective protocol for both the morphological and molecular identification of parasites and may be an easy, cost-effective option that requires little specialized equipment for use in the field. While parasite eggs seem to be largely unaffected in ethanol, we caution researchers to note that deformation of larval tissues may occur, impeding morphological identification for certain species without rehydration^24^. Strategies for mitigating the effects of abiotic factors on parasite preservation should be considered as well and may include ensuring that the entire sample is fixed at the time of collection by entirely submerging, or even homogenizing the sample completely in the preservative, minimizing preservative evaporation by tightly sealing the collection tube with tape or parafilm, and storing the samples in a cool, dry, preferably dark location to regulate temperature and other environmental conditions.

Non-invasive monitoring of parasites in host populations can greatly augment our understanding of host-parasite interactions and disease ecology in natural populations, so refining methods for sample collection, storage and analysis is critical^10,25^. Our results suggest that both ethanol and formalin preserved samples can be used in morphological parasite identification after over a year of storage at room temperature, increasing the accessibility of this practice, especially in resource-limited sites and situations. Ethanol-preserved samples have the added benefit of being able to be subsequently used in molecular studies as well, which can deepen our understanding of parasite genetic and taxonomic diversity while complementing morphological data collected from the same samples. We are optimistic that these mixed-method approaches will contribute significantly to our understanding of global host-parasite dynamics globally in the future.

### Supplementary Information

Supplementary Information.

## Data Availability

All data, including sample lists, R Codes and links to parasite images can be found at: https://github.com/MCHenriquez/ParasitePreservationComparison.
