# Supplementary material for: Assessing morphological preservation of gastrointestinal parasites from fecal samples of wild capuchin monkeys (Cebus imitator) stored in ethanol versus formalin
Source: Sci Rep. 2024 Feb 13;14:3623. doi: 10.1038/s41598-024-53915-2 (PMC10864282; doi:10.1038/s41598-024-53915-2)
Supplement: Supplementary file 1 — Supplementary Information. [file 41598_2024_53915_MOESM1_ESM.pdf]

Title: Assessing morphological preservation of gastrointestinal parasites in ethanol and formalin stored fecal samples collected from wild capuchin monkeys (*Cebus imitator*)

Authors and affiliations: Joelle Hass* 1,2; Megan C. Henriquez* 1,2,3,4; Jessica Churcher 1,2; Hadjira Hamou 1,2; Suheidy Romero Morales 5; Amanda D. Melin* 1,2,6,7 (ORCID: 0000-0002-0612-2514)

1 Department of Anthropology and Archaeology, University of Calgary, Calgary, Alberta, Canada
2 Host Parasite Interactions Network, University of Calgary, Calgary, Alberta, Canada
3 Department of Anthropology, The Graduate Center, City University of New York, New York, NY, USA
4 The New York Consortium in Evolutionary Primatology (NYCEP), New York, New York, USA
5 Área de Conservación Guanacaste, Guanacaste, Costa Rica
6 Alberta Children's Hospital Research Institute, University of Calgary, Calgary, Alberta, Canada
7 Department of Medical Genetics, University of Calgary, Calgary, Alberta, Canada
** Corresponding authors*
Joelle Hass (Joelle.Hass@ucalgary.ca)
Megan Henriquez (mhenriquez1@gradcenter.cuny.edu)
Amanda D. Melin (amanda.melin@ucalgary.ca)

SUPPLEMENTARY INFORMATION

1. Supplementary Table 1
2. Supplementary Figure 1

Supplementary Table 1. Paired fecal samples collected from a wild population of Costa Rican capuchin monkeys and stored in either ethanol or formalin to test the effects of preservation medium on diagnostic morphological identification of gastrointestinal parasites. † Parasites per fecal gram.

SAMPLE NUMBER	MEDIUM	COLLECTION TIME	COLLECTION DATE	DATE SCREENED	TIME ELAPSED (DAYS)	MONKEY	SAMPLE WEIGHT (G)	PARASITE MORPHOTYPE DIVERSITY	PFG†
**PA-662**	Ethanol	07:04	7/6/21	7/14/22	373	Kopa	1.19	3	10.92
**PA-663**	Formalin	07:04	7/6/21	7/7/22	366	Kopa	1.60	7	17.50
**PA-669**	Ethanol	08:14	7/19/21	6/16/22	332	Kenai	2.08	2	139.42
**PA-670**	Formalin	08:14	7/19/21	6/14/22	330	Kenai	3.28	7	289.33
**PA-677**	Ethanol	10:00	7/19/21	9/16/22	424	Lampwick	2.62	2	148.85
**PA-678**	Formalin	10:00	7/19/21	10/28/22	466	Lampwick	3.03	2	131.68
**PA-679**	Ethanol	11:15	7/19/21	6/17/22	333	Badger	1.98	4	4.55
**PA-680**	Formalin	11:15	7/19/21	6/28/22	344	Badger	2.83	6	8.13
**PA-681**	Ethanol	09:20	7/20/21	9/9/22	416	Sully	3.17	3	34.38
**PA-682**	Formalin	09:20	7/20/21	9/16/22	423	Sully	0.90	3	352.22
**PA-687**	Ethanol	07:16	8/10/21	5/10/22	273	Hippogriff	1.70	4	293.53
**PA-688**	Formalin	07:16	8/10/21	5/12/22	275	Hippogriff	2.22	2	55.41
**PA-690**	Ethanol	08:45	8/10/21	6/1/22	295	Petunia	2.24	4	414.73
**PA-691**	Formalin	08:45	8/10/21	5/31/22	294	Petunia	2.57	9	237.74
**PA-692**	Ethanol	09:00	8/10/21	7/5/22	329	ElvisCrespo	1.63	2	636.20
**PA-693**	Formalin	09:00	8/10/21	6/30/22	324	ElvisCrespo	2.00	3	1208.00
**PA-694**	Ethanol	09:49	8/10/21	10/21/22	437	Honeydukes	1.85	3	19.46
**PA-695**	Formalin	09:49	8/10/21	10/21/22	437	Honeydukes	2.00	3	56.00
**PA-698**	Ethanol	07:57	8/11/21	7/21/22	344	Lando	2.30	6	117.36
**PA-697**	Formalin	07:57	8/11/21	7/19/22	342	Lando	3.11	4	64.35
**PA-702**	Ethanol	08:45	8/11/21	9/23/22	408	Cosgworth	2.56	6	80.83
**PA-701**	Formalin	08:45	8/11/21	9/23/22	408	Cosgworth	2.66	4	31.25
**PA-706**	Ethanol	09:28	8/11/21	5/5/22	267	Baloo	2.84	5	272.11
**PA-705**	Formalin	09:28	8/11/21	5/6/22	268	Baloo	2.51	3	106.34
**PA-709**	Ethanol	10:10	8/11/21	7/28/22	351	Appo	2.49	3	113.20
**PA-708**	Formalin	10:10	8/11/21	7/26/22	349	Appo	2.50	4	43.37
**PA-711**	Ethanol	11:30	8/11/21	4/26/22	258	MadMadamMim	3.08	4	3.18
**PA-710**	Formalin	11:30	8/11/21	4/28/22	260	MadMadamMim	4.40	6	25.00
**PA-719**	Ethanol	08:40	8/12/21	8/4/22	357	Sully	1.71	7	11.02
**PA-718**	Formalin	08:40	8/12/21	8/2/22	355	Sully	2.45	2	25.73
**PA-722**	Ethanol	07:33	8/18/21	8/15/22	362	Leviosa	2.07	6	150.35
**PA-721**	Formalin	07:33	8/18/21	8/16/22	363	Leviosa	1.41	1	54.11
**PA-728**	Ethanol	09:30	8/18/21	6/9/22	295	Winky	1.48	7	94.74
**PA-727**	Formalin	09:30	8/18/21	6/7/22	293	Winky	2.66	4	28.38
**PA-744**	Ethanol	11:11	8/19/21	5/18/22	272	Oregano	2.01	4	72.83
**PA-743**	Formalin	11:11	8/19/21	5/17/22	271	Oregano	5.19	4	91.04
**PA-748**	Ethanol	08:30	8/26/21	10/7/22	407	Abu	1.72	6	34.48
**PA-747**	Formalin	08:30	8/26/21	10/7/22	407	Abu	1.45	4	5.23
**PA-754**	Ethanol	11:19	8/26/21	10/14/22	414	Wall-E	1.50	9	71.43
**PA-753**	Formalin	11:19	8/26/21	10/14/22	414	Wall-E	1.40	3	29.33
**PA-761**	Ethanol	08:15	8/30/21	5/26/22	269	Quidditch	2.11	4	225.50
**PA-760**	Formalin	08:15	8/30/21	5/24/22	267	Quidditch	3.02	4	189.57

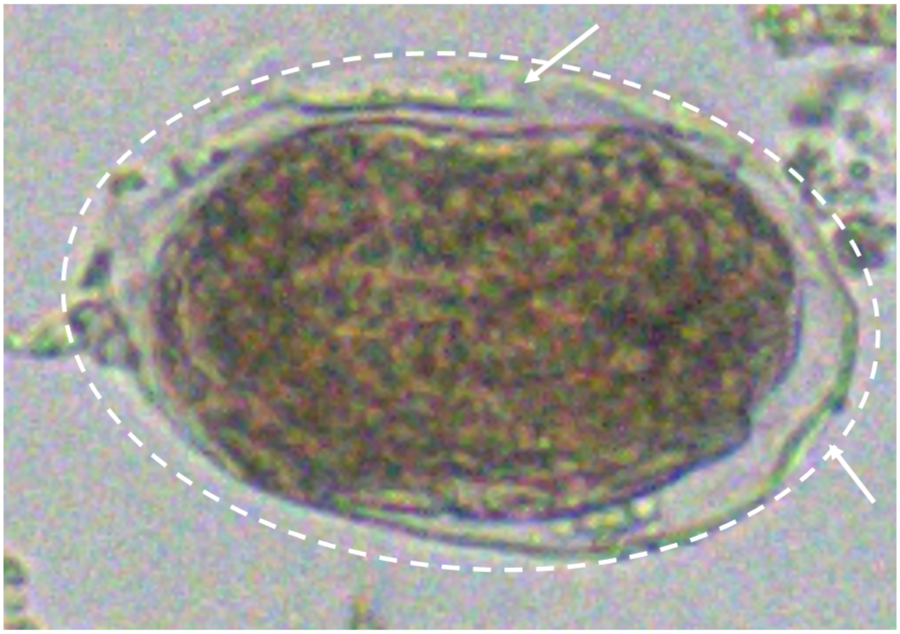

Supplementary Figure 1. A poorly preserved strongyle-type egg (preservation rating 1) from a fecal sample collected from wild capuchin (*Cebus imitator*) monkeys and stored in 96% Ethanol. Note that the shell no longer retains its characteristic oval shape (highlighted by the white dashed line) and shows significant breakage and damage to (white arrows). Embryo within seems unaffected.
